# First step towards a consensus strategy for multi-locus diagnostic testing of imprinting disorders

**DOI:** 10.1186/s13148-022-01358-9

**Published:** 2022-11-07

**Authors:** Deborah Mackay, Jet Bliek, Masayo Kagami, Jair Tenorio-Castano, Arrate Pereda, Frédéric Brioude, Irène Netchine, Dzhoy Papingi, Elisa de Franco, Margaret Lever, Julie Sillibourne, Paola Lombardi, Véronique Gaston, Maithé Tauber, Gwenaelle Diene, Eric Bieth, Luis Fernandez, Julian Nevado, Zeynep Tümer, Andrea Riccio, Eamonn R. Maher, Jasmin Beygo, Pierpaola Tannorella, Silvia Russo, Guiomar Perez de Nanclares, I. Karen Temple, Tsutomu Ogata, Pablo Lapunzina, Thomas Eggermann

**Affiliations:** 1 Wessex Regional Genetics Laboratory, Salisbury, SP2 8BJ UK; 2 Faculty of Medicine, University of Southampton, Southampton, SO17 1BJ UK; 3 Department of Human Genetics, Laboratory for Genome Diagnostics, Amsterdam UMC, Amsterdam, Netherlands; 4 Department of Molecular Endocrinology, National Research Institute for Child Health and Development, Tokyo, Japan; 5 Instituto de Genética Médica y Molecular (INGEMM)-IdiPAZ, Hospital Universitario La Paz, Madrid, Spain; 6 CIBERER- ISCIII and INGEMM, Institute of Medical and Molecular Genetics, Hospital Universitario La Paz, Madrid, Spain; 7 ERN-Ithaca, European Reference Networks, Brussels, Belgium; 8 Rare Diseases Research Group, Molecular (Epi)Genetics Laboratory, Bioaraba Health Research Institute, Araba University Hospital-Txagorritxu, C/Jose Atxotegi s/n, 01009 Vitoria-Gasteiz, Spain; 9 INSERM, UMR 938, Centre de Recherche Saint-Antoine (CRSA), APHP Hôpital Trousseau, Sorbonne Université, 75012 Paris, France; 10 Institute of Human Genetics, University of Hamburg, Hamburg, Germany; 11 Institute of Biomedical and Clinical Science, University of Exeter Medical School, Exeter, UK; 12 Centre de Référence du Syndrome de Prader-Willi et Autres Obésités Avec Troubles du Comportement Alimentaire, Unité d’Endocrinologie, Obésité, Maladies Osseuses, Génétique et Gynécologie Médicale, Hôpital des Enfants CHU Toulouse, Toulouse, France; 13 Laboratoire de Génétique Médicale, Institut Fédératif de Biologie CHU Toulouse, Toulouse, France; 14 Kennedy Center, Department of Clinical Genetics, Copenhagen University Hospital, Rigshospitalet, Copenhagen, Denmark; 15 Department of Clinical Medicine, Faculty of Health and Medical Sciences, University of Copenhagen, Copenhagen, Denmark; 16 Department of Environmental, Biological and Pharmaceutical Sciences and Technologies, University of Campania ‘Luigi Vanvitelli’, Caserta, Italy; 17 Institute of Genetics and Biophysics ‘Adriano Buzzati–Traverso’ CNR, Naples, Italy; 18 Department of Medical Genetics, University of Cambridge, Cambridge, CB2 0QQ UK; 19 Institute of Human Genetics, University Hospital Essen, University Duisburg-Essen, Essen, Germany; 20 Medical Cytogenetics and Molecular Genetics Laboratory, Centro di Ricerche e Tecnologie Biomediche, Istituto Auxologico Italiano, IRCCS, Milan, Italy; 21 Wessex Clinical Genetics Service, University Hospital Southampton, Southampton, UK; 22 Department of Pediatrics, Hamamatsu Medical Center and Department of Biochemistry, Hamamatsu University School of Medicine, Hamamatsu, Japan; 23 Institute of Human Genetics, Medical Faculty, RWTH Aachen University, Pauwelsstr. 30, 52074 Aachen, Germany

**Keywords:** Imprinting disorders, Genetic testing, Multi-locus testing, Unexpected molecular diagnosis, Overlapping phenotypes, Multi-locus imprinting disorder

## Abstract

**Background:**

Imprinting disorders, which affect growth, development, metabolism and neoplasia risk, are caused by genetic or epigenetic changes to genes that are expressed from only one parental allele. Disease may result from changes in coding sequences, copy number changes, uniparental disomy or imprinting defects. Some imprinting disorders are clinically heterogeneous, some are associated with more than one imprinted locus, and some patients have alterations affecting multiple loci. Most imprinting disorders are diagnosed by stepwise analysis of gene dosage and methylation of single loci, but some laboratories assay a panel of loci associated with different imprinting disorders. We looked into the experience of several laboratories using single-locus and/or multi-locus diagnostic testing to explore how different testing strategies affect diagnostic outcomes and whether multi-locus testing has the potential to increase the diagnostic efficiency or reveal unforeseen diagnoses.

**Results:**

We collected data from 11 laboratories in seven countries, involving 16,364 individuals and eight imprinting disorders. Among the 4721 individuals tested for the growth restriction disorder Silver–Russell syndrome, 731 had changes on chromosomes 7 and 11 classically associated with the disorder, but 115 had unexpected diagnoses that involved atypical molecular changes, imprinted loci on chromosomes other than 7 or 11 or multi-locus imprinting disorder. In a similar way, the molecular changes detected in Beckwith–Wiedemann syndrome and other imprinting disorders depended on the testing strategies employed by the different laboratories.

**Conclusions:**

Based on our findings, we discuss how multi-locus testing might optimise diagnosis for patients with classical and less familiar clinical imprinting disorders. Additionally, our compiled data reflect the daily life experiences of diagnostic laboratories, with a lower diagnostic yield than in clinically well-characterised cohorts, and illustrate the need for systematising clinical and molecular data.

## Introduction

Imprinting disorders have a common aetiology in disturbed expression of imprinted genes, but have heterogeneous clinical features affecting growth, development, metabolism, behaviour and lifetime risk of metabolic or neoplastic disease [1, 2]. Their estimated total incidence is ~ 1:3000 live births, but uncertainty remains about their frequency and presentation. Whereas the prevalences of the well-known Prader–Willi syndrome (PWS), Angelman syndrome (AS) or Beckwith–Wiedemann syndrome (BWS) have been established, the prevalence for others is unknown or estimates vary by several-fold. Some imprinting disorders are recently recognised and awaiting clinical delineation, and some presentations are likely under-recognised and under-diagnosed [2, 3].

The allelic expression of imprinted genes is defined epigenetically according to their parent of origin, under the control of imprinting centres (ICs) that contain differentially methylated regions (DMRs) with parent of origin-specific DNA methylation [1]. Imprinted genes are expressed from one parental allele only, and pathology arises when their expression level is altered, by changes in the coding sequences or copy number variants (CNVs) affecting the expressed allele; or by meiotic/mitotic errors (uniparental disomy, UPD (inheritance of an entire chromosome or part of it from the same parent)) or imprinting disturbances altering the representation of the expressed alleles (imprinting defect, epimutation), as either loss or gain of methylation (LOM, GOM) [2, 4]. In some molecular subgroups of imprinting disorders, multiple imprinted loci are affected. These include paternal uniparental diploidy (inheritance of all chromosomes from the same parent), generally presenting as Beckwith–Wiedemann syndrome (BWS), and multi-locus imprinting disorder (MLID) [5–8]. Because imprinting disorders can arise from genetic or epigenetic errors, molecular testing must include both genetic and epigenetic analysis, sometimes spanning multiple imprinted loci, in order to arrive at a confirmed diagnosis [9–11]. In case of MLID, so-called maternal effect variants have recently been identified as causative. These genetic variants affect genes encoding members of the subcortical maternal complex (SCMC) which is involved in the maintenance of the maternal imprint in the oocyte and the early embryo (for review: [12]).

Current diagnostic testing protocols for imprinting disorders reflect international guidelines (e.g. [9, 10, 13, 14]), and in most laboratories, commercial diagnostic kits are employed. Many laboratories diagnose imprinting disorders using MS-MLPA (methylation-specific multiplex ligation-dependent probe amplification), which detects both genetic (CNVs) and epigenetic (DNA methylation) disturbances. While some assays focus on single imprinted loci, others include imprinted loci associated with different imprinting disorders, achieving broader coverage with lower analytic density.

With the increasing use of exome and genome sequencing as first-line investigations for genetic diagnosis, patients with broad categories of clinical features undergo simultaneous testing of relevant genes and the sequencing data are analysed by disease-specific “virtual gene panels”. Such a multi-locus approach for imprinting disorders (simultaneously interrogating multiple imprinted loci) could improve the turnaround, efficiency and cost of diagnosis, but it could potentially detect genetic disturbances at loci analysis of which was not specifically requested by the referring clinician. This could result in incidental and “unforeseen” diagnoses with management or counselling implications that might be welcome, unwelcome, or unclear to the clinician and family. Genome-wide DNA methylation panels have gained recognition as a potentially powerful tool for diagnosing genetic syndromes associated with distinctive genomic DNA methylation patterns (episignatures) [Sadikovic et al. [45], but they are not currently widely adopted for imprinting disorders, perhaps because they are relatively novel, because of cost and accessibility considerations or because of the potential for incidental findings.

Diagnostic laboratories in different nations have different legal and ethical relationships with the clinicians and patients they serve and hence have different approaches to genomic panel testing in general and to multi-locus imprinting testing in particular. We looked at the experience of laboratories using single-gene and multi-locus approaches for molecular diagnosis of imprinting disorders. We aimed to assess whether multi-locus approaches increased diagnostic rate, whether unforeseen diagnoses were made and what issues might result for clinicians and families, and based on this, to propose potential workflows for multi-locus testing of imprinting disorders.

## Results and discussion

Eleven diagnostic laboratories provided data for 16,364 affected individuals tested for the eight imprinting disorders molecularly characterised by different (epi)genetic alterations (Table 1). The rates and subtypes of diagnoses varied between disorders, and also between laboratories for individual disorders. These differences probably reflect the (epi)genetic features of the disorders, as well as variations in clinical referral patterns, diagnostic approaches of laboratories and demographic features of referral populations. Furthermore, some laboratories are (national) expert centres for specific diseases, and therefore, some subgroup data might be distorted. As standard diagnostic testing for imprinting disorders in all contributing centres was based on peripheral blood samples, some cases might have escaped detection due to mosaicism and different sensitivities of the applied tests, a feature which is characteristic for the imprinting defects and upd(11)pat in BWSp. Thus, some results might be false negative, but this ratio is currently indeterminable as systematic studies on the relevance of mosaicism in imprinting disorders are not available and are difficult to conduct. However, the relative detection rates for the already known molecular subtypes generally correspond to published data.

**Table 1:** Detection rates from the different centres for the imprinting disorders associated with aberrant imprinting marks

Laboratory		Aachen/DE^a^	Amsterdam/NL	Essen/DE	Madrid/ES	Milano/IT	Paris/FR	Salisbury/UK	Tokyo, Hamamatsu/JP	Vitoria-Gasteiz/ES	Total	
First-line test	Molecular diagnosis	ME030/ME032 + ME034	ME030/ME032	ME030/ME032; ME034 for research	ME030/ME032 + ME034 + SNParray	ME030/ME032	ASMM-RT qPCR (IC2, MEST, GRB10, MEG3, IG-DMR}	ME030/ME032	MS- pyrosequencing (IC1, IC2, IG-DMR, PEG 10, MEST)	ME030/ME032;ME034 for research	Total number	ratio of molecular subgroups (resolved + unresolved) in the total cohort
SRS												
Referrals (total number)		1164	546	287	348	586	876	388	455	71	4721	
"Expected" molecular diagnoses	IC1 LOM	158	36	27	44	62	97	27	113	8	572	67,6
	11p15CNVs	8	4	1	2	2	NA	1	2	0	20	2,4
	upd(11)mat	0	0	0	3	0	0	1	1	0	5	0,6
	upd(7)mat	37	3	5	9	17	21	10	31	1	134	15,8
Sum "expected" diagnoses		203	43	33	58	81	118	39	147	9	731	
% "Expected" positive diagnoses		17,4	7,9	11,5	16,7	13,8	13,5	8,2	32,3	12,7	15,5	
Unexpected molecular diagnoses	IC2 LOM	3	2	0	4	1	2	0	1	0	13	1,5
	chromosome 7 alterations	3	1	0	1	3	0	0	0	0	8	0,9
	14q32 alterations^b^	10	0	0	8	5	27	5	14	1	70	8,3
	upd(6)mat	2	NA	0	2	1	NA	NA	2	NA	7	0,8
	upd(20)mat	1	NA	0	0	3	NA	NA	6	NA	10	1,2
	PWS	1	NA	0	0	0	NA	NA	2	NA	3	0,4
	upd(11)pat			0			3	1			4	0,5
Sum "unexpected" diagnoses		20	3	0	15	13	32	6	25	1	115	
% total diagnostic yield (expected + unexpected)		19,2	8,4	11,5	21,0	16,0	17,1	11,6	37,8	14,1	17,9	
percentage of unexpected finding among the total findings		9,0	6,5	0,0	20,5	13,8	21,3	13,3	14,5	10,0	13,6	
MLID^c,d^		10	0		NA	0	14	0	3	2	29	5,1

First-line test***		ME030 + ME034	ME030	ME030; ME034 for research	ME030	ME030; ME034 for research	ASMM-RTqPCR (IC1/IC2/M EST, G RB10 /DLK 1/GTL2)	ME030	MS-Pyrosequencing H19 IGF2, IG-DMR, PEG10, MEST)	ME030; ME034 if positive in ME030	Total number	Ratio of molecular subgroups (resolved + unresolved) in the total cohort
BWS												
Referrals (total number)		475	756	400	679	1028	1258	269	169	66	5100	
Expected molecular diagnoses	IC1 GOM	16	16	10	4	17	73	1	15	1	153	11,8
	IC2 LOM	78	84	60	152	184	201	27	32	15	833	64,0
	CNVs 11p	9	5	4	2	7	0	1	2	2	32	2,5
	upd(11)pat	44	39	20	14	83	0	16	32	6	254	19,5
	Of these uniparental diploidy^d^	4	2	1	2		0	1	1	0	11	
Sum "expected" diagnoses		147	144	94	172	291	274	45	81	24	1272	
% "expected" positive diagnose^e^		30,9	19,0	23,5	25,3	28,3	27,8	16,7	47,9	36,4	24,9	
unexpected molecular diagnoses	IC1 LOM	3	0	0	11	3	9	0	0	1	27	2,1
	PHP	0	NA	NA	0	0	NA	NA	2	NA	2	0,2
Sum "unexpected" diagnoses		3	0	0	11	3	9	0	2	1	29	
% total diagnostic yield (expected + unexpected)		31,6	19,0	23,5	27,0	28,6	22,5	16,7	49,1	37,9	25,5	
Percentage of unexpected findings among the total findings		2,0	0,0	0,0	6,0	1,0	3,2	0,0	2,4	4,0	2,2	
MLID^cd^		21	3	3	20	25	22	3	2	8	107	12,8

Laboratory		Aachen/DE	Amsterdam/NL	Essen/DE	Madrid/ES	Milano/IT	Salisbury/UK	Tokyo, Hamamatsu/JP	Vitoria- Gasteiz/ES	Additional specialist laboratories^f^	Total number	ratio of molecular subgroups in the total cohort
First-line test	Molecular diagnosis	disease-specific (MLPA) assays										
PWS^g^												
Referrals (total number)		33	344	825	218	420	400	260	87	420	3007	
Molecular diagnoses	SNRPN ID	4	0	32	2	13	1	9	1	2	64	10,0
	del 15 pat	6	20	59	9	22	15	18	3	100	252	39,4
	upd(15)mat	1	7	48	13	20	10	49	10	0	158	24,7
	dup 15 mat	0	3	6	8		18	2		29	66	10,3
	upd(15)mat/ID unresolved	0	2	32	0	26	5	35	0	0	− 100	15,6
	total	11	32	177	32	81	49	113	14	131	640	
	% positive diagnoses	33,3	9,3	21,5	14,7	19,3	12,3	43,5	16,1	3f,2	21,3	
AS^g^												
Referrals (total number)		17	103	631	131	630	165	49	87	NA	1813	
Molecular diagnoses	SNRPN ID	2	0	77	4	20	0	10	3	116	232	28,5
	del 15 mat	2	6	31	10	88	6	10	7	160	320	39,3
	upd(15)pat	0	2	13	3	12	2	5	1	38	76	9,3
	dup 15 pat	0	3	0	1	1	1	0	0	6	12	1,5
	UBE3A^h^	NA	3	3	1	52	NA	2	3	64	128	15,7
	upd(15)paVID unresolved	0	4	0	NA	6	3	10	0	23	46	5,7
	total	4	18	124	19	179	12	37	14	407	814	
	% positive diagnoses^h^	23,5	17,5	19,7	14,5	28,4	7,3	75,5	16,1	NA	NA	
TS14^b,g^												
Referrals (total number)				144	8	1	73	57	1		284	
Molecular diagnoses	upd(14)mat	NA	NA	3	3	1	11	21	1	NA	40	48,8
	del(14q32)pat	NA	NA	1	1	0	1	6	0	NA	9	11.0
	ID	NA	NA	9	0	0	3	13	0	NA	25	30,5
	upd(14)mat/ID unresolved	NA	NA	1	0	0	2	5	0	NA	8	9,8
	total			14	4	1	17	45	1		82	
	% positive diagnoses			9,7	50,0	100,0	23,3	78,9	100,0		28,9	
KOS14^g^												
Referrals (total number)		NA	NA	8	4	1	8	76	1	NA	98	
Molecular diagnoses	upd(14)pat	NA	NA	2	2	0	3	26	0	NA	33	46,5
	del(14q32)mat	NA	NA	2	0	1	1	9	1	NA	14	19,7
	ID	NA	NA	1	0	0	1	15	0	NA	17	23,9
	upd(14)pat/ID unresolved	NA	NA	0	0	0	3	4	0	NA	7	9,9
	total			5	2	1	8	54	1		71	
	% positive diagnoses			62,5	50,0	100,0	100,0	71,1	100,0		72,4	
_PHp_^g,h^												
Referrals (total number)		NA	NA	NA	4		335	175	239	NA	753	
Molecular diagnoses	upd(20)pat	NA	NA	NA	1		4	0	7	NA	12	2,5
	del(20)mat	NA	NA	NA	0		1	0	8	NA	9	1,9
	ID	NA	NA	NA	0		117	27	58	NA	202	42,6
	upd(20)mat/ID unresolved	NA	NA	NA	0		1	19		NA	20	4,2
	STX16 del	NA	NA	NA	0		34	36	19	NA	89	18,8
	GNAS mutations	NA	NA	NA	0			43	99	NA	142	30,0
	total				1		157	125	191		474	
	% positive diagnoses				25,0		46,9	71,4	79,9		62,9	
TNDM^g^												
Referrals (total number)		NA	NA	12	6	NA	272	6	1	291	588	
Molecular diagnoses	upd(6)pat	NA	NA	0	1	NA	44	2	1	22	70	34,0
	dup(6)pat	NA	NA	2		NA	23	1	0	26	52	25,2
	ID	NA	NA	0	2	NA	45	3	0	16	66	32,0
	of these, ZFP5T	NA	NA	NA	NA	NA	17	NA	NA	7	24	
	upd(6)pat/ID unresolved	NA	NA	0	0	NA	9	0	0	9	18	8,7
	total			2	3		121	6	1	73	206	
	% positive diagnoses			16,7	50,0		44,5	100,0	100,0	25,1	35,0	

(ID imprinting defect (epimutation). NA not assessed/assessable.

^a^the majority of data from Aachen/DE have already been included in [22]]

^b^TS14 overlaps with SRS and PWS and does not have strict clinical criteria

^c^MLID detection rate depends on the tests applied by the laboratory. MLID % given as percentage of individuals with an imprinting anomaly, not as percentage of total referrals.

^d^not (systematically) analysed by all laboratories and not in all patients.

^e^in some laboratories, *CDKN1C* sequencing is included in first-line testing but results are not listed here.

^f^additional specialist centres with expertise in the respective disorders are: Exeter/UK (TNDM); Toulouse/FR (PWS).

^g^Referral for these disorders is biased, *e.g.* because samples were forwarded after exclusion of large deletions (e.g. in case of PWS, AS) or other pretesting steps.

^h^*UBE3A* and *GNAS* sequencing analysis have not been performed in all laboratories. Unresolved: Some MS tests do not discriminate the molecular cause of DNA methylation change: for example, MS PCR does not discriminate between CNV, UPD or imprinting defect; MS-MLPA cannot distinguish between UPD and imprinting defects. Discrimination entails additional tests such as microsatellite analysis or SNP array analysis, but these tests require parental DNA samples and/or allow identification only of uniparental isodisomy. In some patients with PWS, AS, TS14, KOS14 and TNDM, parental DNA samples were not available, additional testing was not performed, and therefore, discrimination between UPD and imprinting defects was not possible. These samples are identified as “unresolved”.)

For clarity, observations from different disorders will be considered separately, in descending order of number of referred samples in the study.

### Silver–Russell syndrome and Silver–Russell syndrome-related phenotypes

The major molecular findings in SRS are LOM of *H19/IGF2*:IG-DMR in 11p15 (SRS-11p) and upd(7)mat (SRS-upd(7)mat); therefore, international guidelines recommend testing of the chromosomes 11p15.5 and 7 DMRs [9]. Thus, many laboratories use two MS-MLPA kits (ME030, ME032) which interrogate DMRs on chromosomes 6, 7, 11 and 14.

In total, 4721 patients had been referred for SRS testing. Interrogating the DMRs of chromosomes 7 and 11 only, diagnostic rates varied from 7.9 to 32.3% (average 15.5%), which may reflect whether clinicians referred only the children meeting clinical thresholds, or referred also for diagnoses of exclusion. Among the positively tested patients (expected and unexpected findings), 67.6% and 15.8% represented *H19/IGF2*:IG-DMR LOM and upd(7)mat, respectively. By first-line testing, CNVs affecting the 11p15.5 DMRs and upd(11)mat were identified as well, but they were rare (2.4% and 0.6%, respectively). Five cases of upd(11)pat were diagnosed, illustrating the occasional challenge of differentiating between lateralised overgrowth (hemihyperplasia) or undergrowth (hemihypoplasia) as a cause of body asymmetry and the value of molecular diagnosis for instituting tumour surveillance [15–17].

Laboratories using the ME032 assay (Table 2) detected further imprinting disturbances in children with SRS features, including LOM of the *MEG3*:TSS-DMR in 14q32 (molecularly corresponding to TS14, SRS-14q) and GOM of the *PLAGL1*:alt-TSS-DMR (e.g. [18, 19]). Laboratories testing for TS14 identified 70 SRS individuals (8.3% diagnostic rate), confirming the value of chromosome 14 testing in SRS referrals. Clinical features of TS14 patients overlap with the features of both SRS and PWS, potentially related to both the age of the patient and the genetic lesion involved [18, 20, 21].

**Table 2:** Imprinted DMRs which should be addressed in (future) multi-locus assays

Imprinted DMR	Chromosome	Imprinting disorder in which the DMR is primarily altered^c^	Physical position (GRCh38/hg38)	Addressed by MS-MLPA assay
*PLAGL1*:alt-TSS-DMR	6q24.2	TNDM upd(6)mat	chr6:g.144006941 -144,008,751	ME032, ME033, ME034
*GRB10*:alt-TSS-DMR	7p12.1	SRS^d^	chr7:g.50781029 -50,783,615	ME032, ME034
*MEST*:alt-TSS-DMR	7q32.2	SRS^d^	chr7:g.130490281 -130,494,547	ME032, ME034
*H19/IGF2*:IG-DMR	11p15.5	SRS, BWS	chr11:g.1997582 -2,003,510	ME030, ME034
*KCNQ1OT1*:TSS-DMR		BWS	chr11:g.2698718 -2,701,029	ME030, ME034
*MEG3*:TSS-DMR^b^	14q32.2	TS14, KOS14	chr14:g.100824187 -100,827,641	ME032, ME034
*MEG3/DLK1*:IG-DMR^a^			chr14:100,811,001–100,811,037	None
*MAGEL2*:TSS-DMR^b^	15q11.2	PWS, AS	chr15:g.23647278 -23,648,882	ME028
*SNURF*:TSS-DMR			chr15:g.24954857 -24,956,829	ME028, ME034
*ZNF597*:TSS-DMR	16p13.3	upd(16)mat	chr16:g.3442828 -3,444,463	None
*PEG3*:TSS-DMR	19q13.43		chr19:g.56837125 -56,841,903	ME034
*GNAS-NESP*:TSS-DMR	20q13.32	PHP upd(20)mat	chr20:g.58838984 -58,843,557	ME031, ME034
*GNAS-AS1*:TSS-DMR			chr20:g.58850594 -58,852,978	ME031, ME034
*GNAS-XL*:Ex1-DMR			chr20:g.58853850 -58,856,408	ME031, ME034
*GNAS A/B*:TSS-DMR			chr20:g.58888210–58,890,146	ME031, ME034

With the exception of *PEG3:*TSS-DMR, all are associated with clinical pictures. The physical positions are based on [47] with the exception of *MEG3/DLK1*:IG-DMR (^a^) which has been determined by [48]. (^b^These DMRs represent secondary DMRs. ^c^In case an imprinting disorder locus comprises several DMRs, the DMRs might be differentially affected by the molecular subtypes. Just internationally accepted imprinting disorders are listed. ^d^These DMRs are affected by (segmental) upd(7)mat or CNVs, but isolated IDs have not yet been described.)

Laboratories performing multi-locus testing made additional diagnoses, including upd(6)mat (*n* = 7), upd(20)mat (*n* = 10) and PWS (*n* = 3). PWS in particular is an important diagnosis for intensive targeted management. The rate of 5.1% of MLID among individuals with *H19/IGF2*:IG-DMR LOM confirmed data from the literature [22], but this number might be an underestimate, as not all patients with *H19/IGF2*:IG-DMR LOM are routinely tested for MLID.

In total, testing for atypical imprinting disturbances on chromosomes 11, 14, 15,and 20 increased the rate of positive diagnoses by 2.5%, with a range of 0.5–5.5%, probably reflecting the range of additional tests performed by laboratories, and the (epi)genetic and/or phenotypic heterogeneity of clinical referrals. This suggests that diagnosis could be streamlined by a multi-locus approach. Ideally, first-line testing would comprise DNA methylation analysis spanning imprinted loci on chromosomes 6, 7, 11, 14, 15, 16, 20. Clinicians referring SRS patients for first-line testing might receive unexpected diagnoses of PWS, BWS or MLID unless specifically opting out to receive secondary diagnoses.

Of note, coding variants in several genes that can give rise to SRS-like presentations (*e.g. IGF2, HMGA2, PLAG1, CDKN1C*) are not represented in this survey.

### Beckwith–Wiedemann syndrome/Beckwith–Wiedemann syndrome spectrum

BWSp is associated with (epi)genetic defects of two imprinted loci on chr11p15 (*H19/IGF2*:IG-DMR/IC1, *KCNQ1OT1*:TSS-DMR/IC2) which must both be analysed in first-line testing as recommended by the international guidelines [16]. In total, 5,100 individuals were referred for BWS testing, with the majority being analysed by MS-MLPA with the chromosome 11p15 assay (ME030).

The diagnostic rate targeting the 11p15.5 loci was 24.9% (range 10.4–47.9%). The variation in diagnostic rate between laboratories may reflect different referral patterns, from diagnosis of exclusion to adherence to international guidelines, or different thresholds for detection and reporting of molecular mosaicism. Prevalence of different molecular diagnoses were broadly in accord with published data: LOM of *KCNQ1OT1*:TSS-DMR (64.0%), upd(11)pat (19.5%) and GOM of *H19/IGF2*:IG-DMR (11.8%) [10].

The majority of patients have either *KCNQ1OT1*:TSS-DMR LOM or upd(11)pat, each of which carries a risk of multi-locus methylation change, either MLID or paternal uniparental diploidy, respectively. Some laboratories tested for MLID in individuals shown to have *KCNQ1OT1*:TSS-DMR LOM, either in routine diagnostic workup or on research basis, and 107 had MLID. The overall rate of MLID was 12.7% but multi-locus tests were not conducted in all patients. Among 254 individuals with upd(11)pat, 11 had paternal uniparental diploidy; this detection rate (4.3%) is probably an underestimate reflecting limited adoption of testing among laboratories [22]. Reported individuals with paternal uniparental diploidy show different neoplasia predisposition from the other molecular subtypes of BWSp, but description of further cases would be valuable to guide targeted management [5, 8, 23, 24]. Patients with uniparental diploidy also have an increased risk of rare recessive disorders resulting from homozygosity for recessive pathogenic variants.

Of note, one laboratory molecularly diagnosed PHP in two cases referred for BWS and showing imprinting defects at both 11p15 and chromosome 20 DMRs [25]. Overgrowth is recognised in both disorders, but overlap between them is little recognised and warrants further consideration.

It should be noted that pathogenic variants in *CDKN1C* significantly contribute to the molecular spectrum of BWS (for review: [10]), but are not considered in this study as *CDKN1C* sequencing is not performed in the first-line workup.

In summary, the data presented here provide an argument for multi-locus analysis not necessarily as first-line testing, but as a secondary test after positive diagnoses of *KCNQ1OT1*:TSS-DMR LOM. As already suggested, patients diagnosed with upd(11)pat have to be tested for paternal uniparental diploidy [10].

### Further imprinting disorders associated with single imprinted loci

#### PWS, AS, TS14, KOS14, PHP, TNDM

For these disorders, multi-locus analysis is not required in the first-line testing, and reflecting this, very limited data are available on MLID or other atypical diagnoses.

*PWS/AS,* which involve the imprinted *SNRPN*/*UBE3A* gene cluster on chromosome 15, were under-represented in this survey compared to their prevalence (both ~ 1:15,000); this may reflect the delivery of PWS/AS diagnostics across many regional centres, compared to the rarer imprinting disorders and national specialist centres audited in this study. As a result, the detection rates for the different molecular subgroups are biased and not representative; in particular, the ratio of 15q11q13 deletions is much lower than expected [26, 27]. Between 5.7 and 15.6% of patients remained without a clear molecular diagnosis in AS and PWS, respectively (designated as “unresolved” in Table 1), due to the lack of parental samples to discriminate between UPD and imprinting defects.

PWS diagnosis is critical for implementing management that transforms clinical outcome [14], and thus, a low clinical threshold is applied for testing, leading to a diagnostic rate of 21.3%. There is some clinical overlap between PWS and TS14 [4], and in one cohort of individuals with PWS features, TS14 was diagnosed as frequently as PWS [21], suggesting that further investigation into a shared diagnostic pathway is warranted. Among patients referred for AS testing, unexpected findings were not commonly observed.

*TS14:* As discussed above, features of TS14 variably overlap with SRS and PWS, with SRS features (SGA, PNGR, relative macrocephaly, feeding difficulties) being more prevalent in infancy and features reminiscent of PWS (hypotonia, tendency to weight gain) increasingly recognisable in childhood. Therefore, direct referral for TS14 was uncommon in this cohort, and the total number of 284 individuals were likely to represent a cohort of patients with mixed phenotypes. As a result, total detection rate of 28.9% is not easy to interpret. However, our study helps to establish the contribution of the different molecular subgroups to the spectrum of molecular TS14, with upd(14)mat as the major group (48.8%), followed by isolated imprinting defects (30.5%) and paternal deletions affecting 14q32 (11%). These proportions resemble those observed in recent papers [28, 29], whereas in early reports imprinting defects and deletions were considerably less frequent than upd(14)mat [18]. This discrepancy probably reflects an ascertainment bias, as the first TS14 patients were carriers of Robertsonian translocations, whereas imprinting defects and deletions were difficult to detect at that time due to methodological limitations.

*KOS14:* Classical clinical features of KOS14 are distinctive, severe and life-shortening, but with increasing recognition of the disorder, less severely affected individuals have been identified [30, 31]. Due to its rarity only 98 cases were audited here, but the diagnostic rate approached 73%: among the resolved cases, 46.5% had upd(14)pat, 19.7% had deletions within the maternal allele, and 23.9% imprinting defects.

*PHP* has relatively specific clinical features, including biochemically measurable abnormalities of calcium, phosphate and parathyroid hormone levels, with variable expressivity of dysmorphisms and bone anomalies [11]. Its prevalence is not known, but it is estimated to be 0.34–1.1 in 100,000 [13]. In our cohort, the total diagnostic rate of 62.9% was lower than the published diagnostic rate which was around 80%, probably because *GNAS* sequencing was not performed by all laboratories, as recommended by the clinical consensus guidelines for inactivating PTH/PTHrP signalling disorders (iPPSD) [13]. Where *GNAS* sequencing was performed, it yielded a diagnostic rate of approximately 30%, which coincides with the previous reports [32]. For the group with methylation alterations, in detail, the majority had imprinting defects (42.6%), followed by *STX16* deletions, causing a *GNAS* A/B hypomethylation in *cis* (18.8%). Paternal upd(20) and larger deletions are rare in PHP.

*TNDM* has an estimated prevalence of 1:300,000, and as such, limited molecular data are available from centres often offering international diagnostic support. The proportions of different molecular diagnoses were in accord with published data [33]. Importantly, 24 of 66 patients with imprinting defects had MLID with biallelic *ZFP57* variants and others had MLID without a *ZFP57* variant. Finding of a *ZFP57* variant has genetic counselling implications, independent of the management and counselling implications of MLID, and therefore, sequencing of this gene and MLID testing is warranted in patients with imprinting defects.

## General discussion

### Clinical and molecular overlap between imprinting disorders

As the symptoms of patients with imprinting disorders often overlap, a specific clinical diagnosis is not always possible. As listed in Table 1, molecular findings characteristic for TS14, PWS and BWS were detected in patients referred for SRS. Among patients with clinical BWSp, molecular findings characteristic for SRS and PHP were detectable, and this overlap was also reported for KOS14 [25, 34]. Furthermore, there are molecular and clinical overlaps between TS14 and PWS, and TNDM and BWS.

Up to now, the majority of patients with MLID exhibit symptoms specific for one of the known imprinting disorders (e.g. BWS and SRS), but there is a growing number of reports on cases with overlapping phenotypes and or epigenotypes or even apparently asymptomatic (e.g. [25]). Thus, it is conceivable that MLID patients currently escape detection as they do not show a distinctive MLID phenotype and are therefore not tested.

In summary, the reports of overlapping and even unexpected molecular findings in patients referred for imprinting disorder testing illustrate that only multi-locus tests enable the detection of this heterogeneous pattern of alterations.

### Imprinted loci to be addressed by future multi-locus imprinting assays

Though more than 10 imprinting disorders have been identified so far, only eight of them are currently known to be associated with molecular disturbances affecting the respective DMR (TNDM, SRS, BWS, TS14, KOS14, AS, PWS, PHP). Therefore, the majority of (multi-locus) test approaches currently target these DMRs, but in the commercially available MLPA kit ME034 two additional DMRs are covered, the *PEG3*:TSS-DMR and the *MEG3*:TSS-DMR. *PEG3*:TSS-DMR is a germline DMR in 19q13.43 without an obvious clinical correlate, but it has been shown to be hypomethylated in the majority of *ZFP57*-associated TNDM imprinting defect patients [35], and also contributes to MLID imprinting signatures. The *MEG3*:TSS-DMR is a secondary (somatic) DMR which is subordinated to the *MEG3/DLK1*:IG-DMR in 14q32; the molecular features of this DMR make its methylation difficult to measure, but the *MEG3*:TSS-DMR acts as a reliable proxy, allowing the unambiguous detection of 14q32 alterations in routine diagnostics. The *ZNF597*:TSS-DMR in 16p13.3 is not targeted in routine testing, but there is increasing evidence suggesting that this DMR is suitable for diagnostic use.

Several studies indicate that further imprinted loci are affected by MLID, but their relevance is currently being discussed (for review: [36]). Thus, future multi-locus tests should comprise flexible formats, but for diagnostic application they should target only imprinted loci for which associations with clinical phenotypes have been established.

### Multilocus Imprinting Disturbances (MLID) testing and its relevance

Multi-locus imprinting disturbances (MLID) affect an unknown subset of individuals with DNA methylation anomalies. While a multi-locus testing strategy is potentially warranted for many imprinting disorders, its implementation would inevitably result in increased detection of MLID. Current consensus guidelines do not recommend MLID testing because its clinical consequences remain uncertain, but MLID is increasingly reported with *trans* acting gene variants in SCMC encoding genes that carry inherent risks of recurrence as well as parental reproductive difficulties [11, 37–39] making a case for testing in families with multiple affected pregnancies [40]. Currently there is insufficient information to confidently establish counselling or management guidelines for MLID, but its intrinsic heterogeneity indicates that information can be gathered only with a concerted international effort.

### New imprinting disorders?

With the increasing application of multi-locus tests for research or diagnostic purposes in cohorts of patients with imprinting disorders, maternal UPDs of chromosomes 6 and 16 have been identified in a growing number patients with intrauterine growth retardation and/or short stature.

In laboratories addressing the *PLAGL1*:alt-TSS-DMR in 6q24 in the patient cohorts, upd(6)mat is a rare but recurring finding, and an association with (intrauterine) growth retardation is meanwhile well established [19]. Thus, identification of upd(6)mat in a patient with a phenotype reminiscent for SRS can be regarded as molecularly diagnosed.

Since its first description in 1993 [41], upd(16)mat has been reported in numerous patients. Carriers of upd(16)mat exhibit a heterogeneous spectrum of features, and therefore, it has been suggested that cell lines with trisomy 16 had an impact on the phenotypic outcome [42]. The recent report on an isolated methylation defect at the *ZNF597* locus [43] provides further evidence for the existence of a 16q13.3 associated imprinting disorder. Testing of the *ZNF597*:TSS-DMR is currently not performed systematically but should be considered in future multi-locus assays.

A third genetic constitution which needs future awareness is paternal UPD of chromosome 7 (upd(7)pat) which has been suggested to be associated with tall stature [44]. The application of multi-locus testing in overgrowth/BWS cohorts will further enlighten the relevance of this alteration.

## Suggestions

**Fig. 1:**
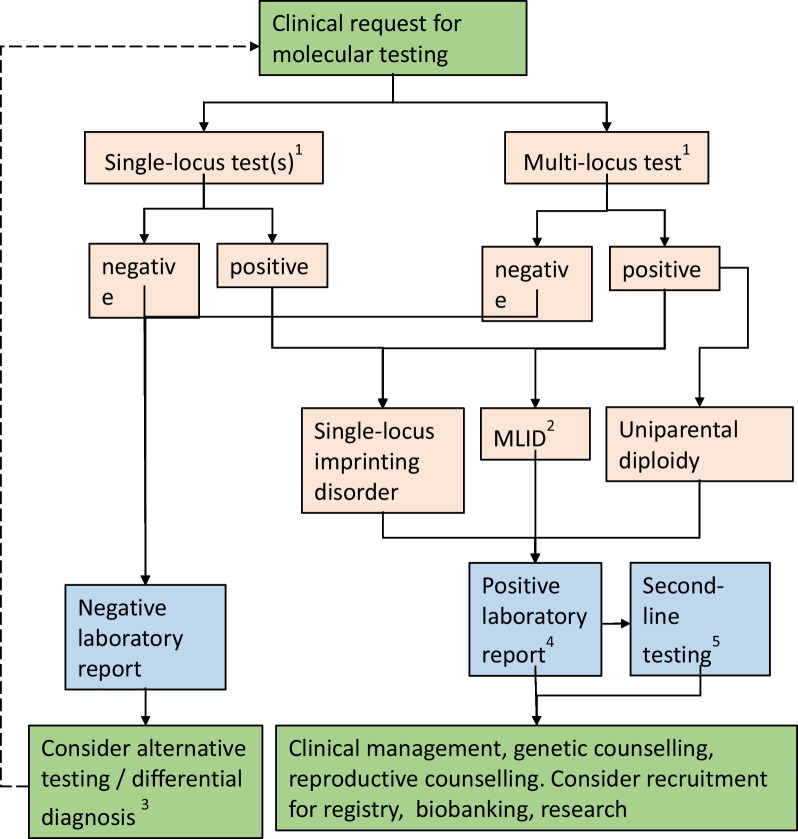
Suggested multi-locus testing algorithm for imprinting disorders. ^1^The decision on first-line test depends on the clinical phenotype of the patient, consensus guidelines and national regulations. For some disorders and phenotypes, single-locus testing might be preferred; for some clinical indications (e.g. relatively non-specific growth restriction, hypotonia or developmental problems or features characteristic of more than one imprinting disorder) multi-locus testing may be preferred. Reproductive and family history may also be considered. ^2^MLID testing should be considered in case of clinical features reminiscent for SRS, BWS, TNDM and PHP. ^3^Differential diagnosis or alternative testing may include NGS-based genomic medicine, microarray, testing of alternative tissues or additional epigenetic analysis, depending on the clinical features of the patient. ^4^Depending on the disorder, national regulations and clinical consensus guidelines, positive reports may also include recommendations for further action such as additional analyses to identify the underlying molecular cause (e.g. discrimination between UPD and ID, exclusion of a Robertsonian translocation in case of a UPD for PWS, AS, TS14, KOS14) to estimate the recurrence risk, clinical management and counselling. ^5^Second-line testing may include NGS-based genomic analysis, detection of *cis* acting SNV or CNV, detection of *trans* acting variants, or other analyses pursuant to relevant consensus guidelines or the molecular change detected

Based on comprehensive datasets from eleven laboratories, our survey shows the evolving nature of imprinting disorder diagnosis. We suggest the following modifications to diagnostic testing for imprinting disorders (Fig. 1):

*Development or adoption of a multi-locus imprinting test*, capturing DNA methylation and relevant copy number analysis of imprinted loci on chromosomes 6, 7, 11, 14, 15, 16, 20. This could conveniently be compassed on one two-tube MS-MLPA kit, or in a novel test which should be validated in respect to a uniform and standardised coverage of all relevant DMRs and major molecular subtypes.

*Multi-locus testing of all individuals referred for growth restricting imprinting disorder testing* (i.e. for SRS, TS14). This would efficiently capture known imprinting disorders and MLID, after which SNP array or NGS (next generation sequencing) analysis could be targeted for individuals; (a) with unusual DNA methylation patterns, to seek uniparental diploidy or structural variants, (b) with MLID to investigate *trans* acting variants in maternal effect genes; (c) without a detected imprinting change to seek variants in genes involved in growth restriction.

*Multi-locus testing for individuals with BWS due to upd(11)pat or IC2 LOM*. This strategy has already been suggested [10] as it enables identification of paternal uniparental diploidy as the basis for a modified neoplasia surveillance. In patients with *KCNQ1OT1*:TSS-DMR LOM patients, it allows detection of MLID. In families where MLID is detected, genetic testing for pathogenic variants in maternal effect genes might be considered.

*Multi-locus testing for individuals with TNDM caused by PLAGL1 LOM.* This would identify MLID enabling genetic testing for either *ZFP57, ZNF445* or variants in maternal effect genes.

Furthermore, we suggest that additional translational studies in a diagnostic setting would be very helpful to resolve several gaps in understanding of imprinting disorders and address unmet needs for counselling and management of affected individuals:

*Multi-locus analysis for individuals negatively tested for TS14 and PWS* might confirm evolving evidence for molecular and clinical overlaps between TS14 and PWS. In these patients, the clinical overlap should be assessed.

*Qualitative study on the ethical acceptability* and the significance of multi-locus testing for clinicians and families.

*Comprehensive genetic, epigenetic and clinical profiling of individuals with MLID in an international cohort,* to improve understanding and clinical management of this condition.

*International and national patient registries for rare imprinting disorders* and exchange of information, to estimate prevalence, determine clinical history and underpin improvements in diagnosis and management.

*Further assessment of comprehensive * [36]* and genome-wide DNA methylation * [45]* testing* for first-line diagnosis or MLID testing in imprinting disorders, including clinical utility, economic viability and ethical acceptability.

## Outcomes

The authors of this study have consented to the following definitions and agreements in the context of multi-locus testing and MLID, as a precursor to future cooperative and international guidelines and research projects:

1. For the purposes of genomic medicine, MLID is defined as: imprinting defects at two or more of the ICs listed in Table 2. A conservative definition of MLID is preferable for use in clinical service, since it focuses on loci that are well studied and clinically relevant.
2. However, imprinting is not biologically restricted to clinically relevant ICs, and current research suggests an alternative, expanded definition: MLID comprises imprinting defects at (A) ≥ 1 clinically relevant loci and (B) ≥ 2 additional (germline) ICs. Further research is required to clarify exactly which ICs comprise group B and whether the expanded definition has diagnostic utility.
3. MLID is a spectrum disorder: its definition is epigenetic, not clinical, and affected individuals are clinically heterogeneous. However, the majority of patients are currently recognised because of a “primary presentation”—clinical features aligning closely with one of the specific imprinting disorders. Though this may reflect ascertainment bias, further studies of patient cohorts with phenotypes that are not currently associated with classical imprinting disorders will provide further insights into epigenotype–phenotype relationships in MLID.
4. The definition of MLID focuses on ICs rather than on secondary DMRs under their control. An example for MLID is loss of methylation (LOM) of both *H19/IGF2*:IG-DMR/IC1 and *KCNQ1OT1*:TSS-DMR/IC2 in 11p15.5 because they are independent germline ICs.
5. In principle, imprinting disturbance may be manifested as LOM / GOM in MLID. In practice, at present, LOM is observed in the majority of cases.

## Perspectives

The precise identification of (new) genetic and epigenetic pathways offers the potential for new therapeutic regimes as the basis for a more directed and personalised treatment in IDs. A diagnosis may alter clinical management, for example of puberty in TS or cancer surveillance in BWS; or change counselling, e.g. when *cis* or *trans* acting genetic variants are identified. On a translational level, new diagnoses of rare disorders such as rare UPDs and MLID will clarify (epi)genotype–phenotype relationships and management.

In the future, methodological progress in methylation specific next and third generation sequencing techniques will allow to target genome-wide genomic alterations (SNVs and CNVs) as well as epigenetic signatures in the same assay (for future perspectives see also [46]). These assays will enable the integrated analyses of genomic and epigenetic data, and in combination with additional *omic* techniques, the causes of disturbed imprinting and their functional consequences will be determined. These single unified tests will avoid false negative results which are currently obtained by focusing on single loci, and will even make the detection of multiple (epi)genetic pathogenic variants with an impact on the phenotype of a patient possible.

## Methods

Eleven diagnostic laboratories from seven countries (Table 1) contributed to this study, providing data from 16,364 patients referred for diagnostic testing for Silver–Russell syndrome (SRS), Beckwith–Wiedemann syndrome (BWS), Prader-Willi syndrome (PWS), Angelman syndrome (AS), Temple syndrome (TS14), Kagami–Ogata syndrome (KOS14), Pseudohypoparathyroidism (PHP) and Transient Neonatal Diabetes Mellitus (TNDM).

The participating centres followed the international diagnosis guidelines when available [9, 10, 13, 14]. While the majority of participating institutions used disease-specific MS-MLPA assays as first-line tests, manufactured by MRC Holland (Amsterdam, the Netherlands) (Table 2): ME028 for PWS/AS, ME030 for BWS/SRS, ME031 for PHP, ME032 for TNDM, TS14, KOS14 and SRS (upd(7)mat), some used other test systems (such as MS pyrosequencing; allele-specific methylated multiplex real-time quantitative PCR (ASMM-RT qPCR)). The diagnostic testing was mainly based on peripheral blood samples; in single cases, buccal swab DNA was analysed.

Many laboratories used further methods (e.g. SNP array analysis, microsatellite analysis/short tandem repeat typing, Sanger sequencing) to confirm the positive findings and/or to further discriminate molecular subgroups. Some centres applied a multi-locus test, e.g. MS-MLPA (ME034), MS pyrosequencing or ASMM-RT qPCR to confirm positive results of first-line testing and/or to identify further molecular changes and MLID. Some laboratories used multi-locus testing routinely for all the referred individuals (Aachen/Germany; Madrid/Spain; Tokyo, Hamamatsu/Japan).

The clinical reasons for referral varied between centres, depending on national practice and (scientific) focus. In some centres/countries, patients with even discrete features of the respective imprinting disorders were referred for testing, while others had relatively strict criteria. Some (national) expert diagnostic centres performed a restricted range of testing for a specified subset of clinical presentations and/or disease loci.

The authors want to emphasise the use of the recently suggested nomenclature of DMRs based on the Human Genome Variation Society (HGVS) guidelines (Table 2)[47].

## Data Availability

The datasets generated and/or analysed during the current study are not publicly available due to privacy restrictions but are available from the corresponding author on reasonable request.

## Abbreviations

ASMM-RT qPCR: Allele-specific methylated multiplex real-time quantitative PCR
BWS: Beckwith–Wiedemann syndrome
BWSp: Beckwith–Wiedemann syndrome spectrum
CNV: Copy number variant
DMR: Differentially methylated region
GNAS: *GNAS-NESP*:TSS-DMR; *GNAS-XL:*Ex1-DMR;*GNA**S **A/B*:TSS-DMR; *GNAS-AS1*:TSS-DMR (20q13)
GOM: Gain of methylation
IC: Imprinting centre
ID: Imprinting defect
iPPSD: Inactivating PTH/PTHrP signalling disorder
KOS14: Kagami–Ogata syndrome
LOM: Loss of methylation
MLID: Multi-locus imprinting disturbance
MLPA: Multiplex ligation-dependent probe amplification
MS: Methylation-specific
NGS: Next generation sequencing
PHP: Pseudohypoparathyroidism
PNGR: Post-natal growth retardation
SCMC: Subcortical maternal complex
SGA: Small for gestational age
SNP: Single-nucleotide polymorphism
SNV: Single-nucleotide variant
SRS: Silver–Russell syndrome
TNDM: Transient neonatal diabetes mellitus
TS14: Temple syndrome
UPD: Uniparental disomy
